# Biology of *Pseudacteon* Decapitating Flies (Diptera: Phoridae) That Parasitize Ants of the *Solenopsis saevissima* Complex (Hymenoptera: Formicidae) in South America

**DOI:** 10.3390/insects11020107

**Published:** 2020-02-06

**Authors:** Li Chen, Sanford D. Porter

**Affiliations:** 1 College of Life Science, Hebei University, Baoding 071001, China; 2 State Key Laboratory of Integrated Management of Pest Insects and Rodents, Institute of Zoology, Chinese Academy of Sciences, Beijing 100101, China; 3 Center for Medical, Agricultural and Veterinary Entomology, United States Department of Agriculture—Agricultural Research Service, Gainesville, FL 32608, USA; sdporter22@gmail.com

**Keywords:** parasitoid, phorid, social insect, *Solenopsis invicta*, red imported fire ant, natural enemies

## Abstract

*Pseudacteon* flies (Diptera: Phoridae) parasitize individual ant workers, causing decapitation of the host during pupariation. Phorid flies that attack South American fire ants in the *Solenopsis saevissima* (Smith) complex are distributed across a wide range of habitats and climates associated with the geographical range of their hosts. Sympatric species sharing the same hosts often partition niche resources by season, active time of day, host size, and/or different host activities. They have the potential of being used for biological control of the imported fire ants in North America, Australia, and Asia.

## 1. Introduction

*Pseudacteon* decapitating flies are specific parasitoids of ants [1]. At least 22 species of *Pseudacteon* flies are known to attack South American fire ants in the *Solenopsis saevissima* (Smith) complex [2,3,4]. Seventeen species are parasitoids of the black imported fire ant, *Solenopsis richteri,* and the red imported fire ant, *Solenopsis invicta* [2]. These fly species are specific to *S. saevissima* complex fire ants and do not occur in populations of *Solenopsis geminata* complex fire ants native to North and Central America [2,5,6,7]. Under forced laboratory conditions, some of these fly species (e.g., *Pseudacteon curvatus*, *Pseudacteon obtusus*, *Pseudacteon tricuspis*, and *Pseudacteon wasmanni*) can parasitize fire ants in the *geminata* complex; however, rates of successful parasitism are always very low [8,9,10,11]. A few flies of *Pseudacteon pradei* and *P. wasmanni* were observed attacking *S. geminata* workers in trays in the field, but these attacks did not result in successful parasitism [9,12]. First-generation flies reared from *S. geminata* complex ants in laboratory still maintained a strong preference for their normal *S. saevissima* complex hosts upon emergence [11]. Extensive field observations and forced laboratory tests have demonstrated that decapitating flies which parasitize *Solenopsis* fire ants are not capable of parasitizing ants in other genera [10]. Six highly specific phorid fly species in the genus *Pseudacteon* from native South America have been successfully released in the USA for biological control of the imported fire ant [13]. In this review, we focus on life history, distribution, phenology, and activity patterns of parasitic *Pseudacteon* flies, and their association with fire ants.

## 2. Parasitic Life of *Pseudacteon* Phorid Flies

**Figure 1:**
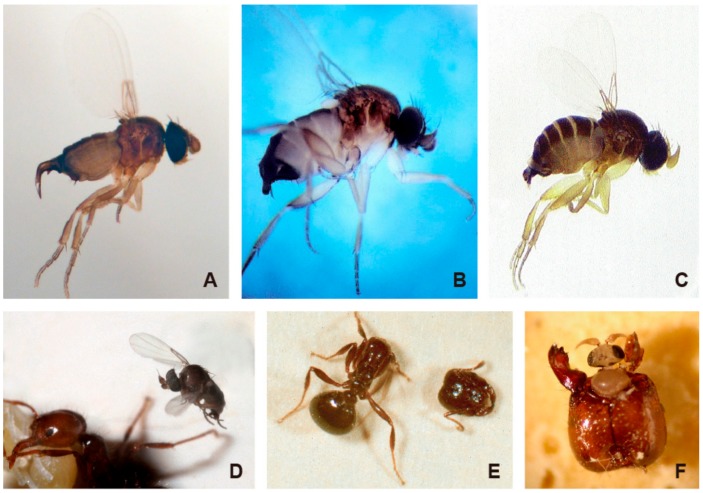
Decaptating phorid flies with specialized ovipositors and their parasitic life cycle. (**A**) *Pseudacteon curvatus* female; (**B**) *Pseudacteon nocens* female; (**C**) *Pseudacteon obtusus* female; (**D**) Female *Pseudacteon litoralis* fly preparing to inject an egg into the thorax of a fire ant worker; (**E**) Fire ant worker decapitated by a fly maggot just prior to pupariation; (**F**) A *P. litoralis* male fly emerging from the head capsule of a parasitized fire ant worker.

Decapitating flies in the genus *Pseudacteon* (Diptera: Phoridae) are koinobiontic/solitary parasitoids of individual worker ants [14]. The parasitic lives of phorid flies that parasitize *Solenopsis* fire ants have been well documented [6,7,10]. The female fly hovers several millimeters above fire ant workers and injects an egg in a rapid aerial attack (<1 s) into the thorax of an appropriate worker with a specialized ovipositor (Figure 1A–D). After hatching, the first-instar larva develops in the thorax and remains inside its serosa until molting into second instar. About four days after attack, the second-instar larva migrates to the head. The third-instar lava proceeds to pupation after consuming all the tissue inside the head capsule and eventually killing the worker [15]. The late third-instar larva appears to release an enzyme that causes the host ant head to fall off (Figure 1E) [16]. Within a few weeks of pupation, the adult fly emerges and crawls out of the decapitated head capsule (Figure 1F). The total developmental time from egg to adult is between 4 and 10 weeks, depending on temperature, host species, host size, and the *Pseudacteon* species (Table 1). Development of phorid flies is accelerated at higher temperatures. The increase in developmental rate ranges from 2.2% to 10.9% per degree within the 20–30 °C range [17]. However, laboratory studies indicate that larval and pupal stages may have lower survivorship at high temperatures [17,18,19,20].

*Pseudacteon borgmeieri*, *Pseudacteon cultellatus*, *P. curvatus*, *Pseudacteon nudicornis*, *Pseudacteon nocens*, *P. obtusus*, and *P. tricuspis* are able to successfully develop in both S. invicta and S. richteri [17,18,20,21,22]. Other phorid species may attempt to oviposit on other saevissima complex fire ants such as Solenopsis macdonaghi and Solenopsis quinquecuspis, but the success of these attacks has not been confirmed [21].

Newly emerged flies are ready to mate and repeat the attack cycle within several hours of eclosion [7]. As completely starved flies rarely live beyond one day, provision of water increases longevity by two days in the laboratory [24]. Sugar feeding can further increase the longevity considerably depending on rearing temperature and fly activity [24,25,26]. The ability to digest monosaccharide sugars (fructose, glucose) and disaccharide sugars (sucrose, trehalose) may explain significant but modest increase in longevity of *P. tricuspis* feeding on cotton aphid honeydew [27,28]; therefore, phorid flies can be expected to feed on nectar and/or honeydew sources in field.

The number of eggs produced by a female *Pseudacteon* varies from about 200 to almost 300 [29]. Female flies attack at a rate of several workers per minute under laboratory conditions and can last for an hour or more with unlimited hosts [6]. Females may attack more frequently if potential hosts are abundant. However, some attacks are not successful. Only 11–35% of oviposition attempts result in successful parasitism and larval development in laboratory tests [6]. In native Argentina, the maximum parasitism rate per *S. invicta* colony by phorid flies is around 2.8% [30]. The low success rates may be attributed to the following possibilities: (1) oviposition strikes are too rapid to eject an egg, (2) flies are selective to quality of workers for egg laying through oviposition attempt, and (3) some of the oviposited eggs do not develop due to infertility or ant defenses [16].

*Pseudacteon* species with different sizes prefer different sizes of fire ant workers [31,32]. In some larger *Pseudacteon* species like *Pseudacteon litoralis*, *P. nocens*, *P. obtusus*, and *P. tricuspis*, male flies are smaller than female flies from the same species, but their size ranges may overlap to some degree [17]. Sex ratio of these flies is thought to be determined by the body size of the host with larger ants producing more females and smaller ants producing more males [33]. However, sexual size dimorphism in smaller fly species like *P. cultellatus* and *P. curvatus* does not appear to occur [17,20,34]. There are no gender differences in host sizes used in *P. cultellatus* [20] and *P. curvatus* [17]. Host size distributions in *P. obtusus* and *P. nocens* were predominately overlapping, but female flies did not develop in the smallest ants and males did not develop in the largest ants [17,18]. In one field test, *P. curvatus* and *P. tricuspis* preferred monogyne fire ant colonies, and more offspring are produced from monogyne than polygyne fire ant colonies, possibly due to poor adaption to polygyne fire ants, which are far less abundant than monogyne ones in South America [34,35]. Because worker size in polygyne form is smaller than in monogyne form [36], more male flies are expected to be produced from the polygyne form [37]. Sex ratios in *Pseudacteon* flies do not appear to be affected by environmental conditions [33].

## 3. Distribution

The geographic distribution of *Pseudacteon* flies in South America has been extensively sampled in the past two decades [3]. As host-specific parasitoids, the geographical range of these flies is largely determined by the range of their hosts, in addition to climatic factors and habitats [38]. The *S. saevissima* complex fire ants occur in vast areas from the Amazon Basin of Brazil, west to the Andes and south to ≈42° S latitude in the Río Negro province, Argentina [39,40]. Early investigations showed that the range of *S. richteri* was southernmost Brazil (Rio Grande do Sui), Uruguay, and northern Argentina and that *S. invicta* occupied a 3000 km long, relatively narrow band centered on the headwaters of the Paraguay River, northward into the Amazon drainage along the Guapore River, and southward into Argentina and Paraguay [41,42,43]. *Solenopsis richteri* extended from approximately 30° S to 38° S in South America, whereas *S. invicta* extended from 10° S to 33° S between Porto Velho, Rondonia Territory, Brazil and Rosario, Argentina [42,44]. The two species are parapatric in their native South America and do not hybridize apparently even where their ranges overlap [44,45]. Their native ranges overlap only in a small area (southern Santa Fe Province, near Rosario) of central Argentina [44]. A large overlapping area seems likely because the southern boundary of the predicted possible range expansion for *S. invicta* matches the current southern range limit of *S. richteri* in South America [46]. While *S. invicta* occupies the tropical and subtropical areas, *S. richteri* occupies the more temperate ecological niche. Consequently, *Pseudacteon* flies follow the distribution of their hosts and are broadly distributed across a wide range of climates and habitats.

Fire ants with broader distributions often have *Pseudacteon* species with wider ranges [38]. Furthermore, a fly species using multiple hosts appears to have larger geographical ranges. In South America, *P. cultellatus* and *P. obtusus* have the broadest distributional ranges of the *Pseudacteon* species associated with *S. saevissima* complex [38]. These two flies attack many common South American fire ants including *Solenopsis interrupta*, *S. invicta*, *S. macdonaghi*, *S. richteri*, and *S. saevissima* [3,21]. Their ranges stretch from subtropical to temperate climates and habitats from Brazil to Argentina [38]. *Pseudacteon obtusus* occurs at the highest altitude (2280 m), the southernmost latitude (Corralito, Río Negro, Argentina, 40°43.786′ S), and the westernmost longitude (Santa Cruz, Tucumán, Argentina, 65°46′ W) [47]. *Pseudacteon curvatus*, *P. litoralis,* and *P. tricuspis* are the most abundant and common species in Argentina and Paraguay [47]. *Pseudacteon curvatus* and *P. nocens* are very abundant and widespread in northern Argentina in varying climatic conditions but rare in Brazil [3,38,47]. *Pseudacteon* species with narrower distributions could be more host-limited, utilizing single hosts with smaller ranges. *Pseudacteon bulbosus,* which paratisizes *S. interrupta* and a species close to *Solenopsis electra*, is found only in the Santiago del Estero province in Argentina [47,48,49]. *Pseudacteon bulbosus* and *Pseudacteon comatus* were often extremely rare in several extensive surveys [47,48,50]. The range of *Pseudacteon conicornis* is known only from *S. saevissima* along the Atlantic coastline of Brazil and appears to be restricted additionally by climate or habitat [3]. The known westernmost record for *Pseudacteon* fly species occurring in South America is a new species morphologically similar to *P. obtusus* (Bulnes, Bio Bio, Chile, 36°52.389′ S, 72°19.659′ W) that attacks *Solenopsis gayi* in central Chile ([40], LA Calcaterra, personal communication).

*Pseudacteon* species are active at 16–37 °C, 20–90% RH, and 0–11.6 km/h wind speed [47]. Those flies with broader geographical distributions appear to have greater climatic (very dry and with extreme temperatures) tolerance [38]. *Pseudacteon bulbosus*, *P. curvatus*, *P. litoralis*, *P. nocens*, *Pseudacteon obtusitus*, and *P. tricuspis* were found to be active under greater climatic stress [47].

Local *Pseudacteon* communities typically consist of multiple fly species. Argentina has higher species diversity than the nearby countries [47]. Researchers have found 13 sympatric fly species attacking *S. interrupta* at sites in northern Argentina, 10 different species attacking *S. invicta* in northeastern Argentina, and 7 species attacking *S. richteri* in eastern Argentina [47,48]. *Pseudacteon cultellatus*, *P. curvatus*, *P. litoralis*, *P. nocens*, *P. obtusus*, and *P. tricuspis* are often codominant species [47,48]. *Solenopsis saevissima* (north climate) and *S. invicta* (south climate) have a parapatric distribution in the state of São Paulo, Brazil, apparently determined by the climate. Two different communities of decapitating flies were associated with *S. saevissima* in the north and with *S. invicta* in the south [51]. The most abundant species in the northern community were *Pseudacteon affinis*, *P. cultellatus*, *Pseudacteon dentiger*, *Pseudacteon disneyi*, and *Pseudacteon fowleri*, and in the southern community were *P. litoralis*, *P. pradei*, *P. tricuspis*, and *P. wasmanni*. The community structure of these flies largely depends on niche partitioning of common host resources. The rich phorid guild found in South America is apparently associated with species diversification of their host complex.

## 4. Phenology and Activity Pattern

Most phorid fly species are active throughout the year in their native range but with different seasonal and daily activity patterns. The abundance of flies in South America is generally high in spring, possibly due to significant host colony activity caused by the mating flights and/or because of the start of the rainy season [30,52]. The peak abundance varies with fly species throughout the year. *Pseudacteon curvatus* reaches peak abundance in summer, *P. comatus*, *P. nudicornis*, *P. obtusus*, and *P. tricuspis* in fall, and *P. borgmeieri* in winter [47,50]. In winter months, there are fewer species and lower abundance, and most fly species disappear during cold winter [50]. Furthermore, the abundance patterns of phorid flies usually vary among different areas. For instance, six species of *S. invicta*-decapitating flies were found active throughout the year in Corrientes [30], whereas only one species of *S. richteri*-decapitating fly was active throughout the year in Buenos Aires [50]. The warmer winter in Corrientes explains greater phorid activity throughout the year. Overall abundance for a given species can be high in spring and fall in northeastern Argentina and in summer in east central Argentina [47]. In Argentina, at least six common fly species, *P. cultellatus*, *P. litoralis*, *P. nocens*, *P. obtusitus*, *P. obtusus*, and *P. tricuspis*, are abundant and present in each month [30,48]. As they oviposit all year round in a temperature range of 18–40 °C, these flies have continual overlapping generations [5]. There can be multiple annual peaks of abundance. The fluctuations in abundance of most fly species can be interpreted, at least partially, by climatic variables related to temperature, moisture, and rainfall because some species are apparently favored by high humidity or warm temperatures [30,50,53]. In addition to environmental factors, the complex seasonal patterns of fly abundance may also depend on genetically based intrinsic physiology, host species, and habitat [30].

Fly species exhibit different activity patterns throughout the day. Host ants can be active all day and night, but *Pseudacteon* parasites are only active during the day. In some areas, fly abundance reaches high level at dusk, but species diversity reaches high level around midday. *Pseudacteon affinis*, *P. disneyi*, *P. litoralis,* and *P. nocens* are usually active in the early morning and late afternoon (more temperate species), whereas *P. cultellatus*, *P. curvatus*, *P. dentiger*, *P. nudicornis*, *P. obtusitus*, *P. obtusus,* and *P. tricuspis* are mainly active during the middle of the day [47,53,54]. Sometimes fly species (e.g., *P. litoralis*, *P. nocens* and *P. obtusus*) can be found attacking ants from morning until dusk in cool season [47,48,50]. Daily activity of phorid flies can be partially explained by climatic variables such as mean temperature, relative humidity, and light intensity. The influence of light intensity on fly occurrence is always associated with temperature changes. At low temperatures, greater numbers of flies are in the sun, whereas at higher temperatures, greater numbers of flies are in the shade. Approximately one-half of the species are found most commonly in full sun and the other half in full shade [55,56]. It seems reasonable that the relative abundances of phorid flies associated with shade are related to the overall effect of solar radiation on the microhabitat rather than to light preference.

Diversified sympatric species sharing the same host resources partition niche space by season and time of day. The species richness of phorid flies may increase gradually in summer time. The seasonal and daily activity patterns of phorid fly match its host activity patterns and are also constrained by the host’s activities. The activity amplitudes of flies correlate to periodic ant foraging activity, i.e., host availability. The ant foraging activity generally increases over the course of the day, and species richness and abundance of phorid fly increase synchronically. Both *Pseudacteon* flies and their ant hosts tune their life cycles to seasonally changing abiotic conditions [50].

## 5. Phorid Fly–Fire Ant Association

A wide array of phorid species can be found attacking *S. invicta* and other *saevissima* complex fire ants [47,48,50,55]. Multiple *Pseudacteon* species in sympatric communities attack almost all sizes of fire ant workers in nature [31,32]. The coexistence of multiple fly species on a single host resource suggests that these flies may partition ecological niches with time of day or season, different host sizes, and attraction to different host locations [37,48,50,57]. In the presence of phorids, *S. invicta* workers usually adopt different foraging strategies like covering food resource with debris and dirt, foraging in tunnel systems, and/or increasing nocturnal activity. Some Brazilian colonies show a strong freezing response to the presence of phorid flies. The hiding behavior may allow fire ants to regain control of large food resources when the flies leave [58]. The presence of phorids, however, can also lead to turnover of resources from host species to competing ant species by reducing its competitive dominance in competitive interactions [59]. Phorid parasitism pressure is considered to be responsible for these behavioral adaptions.

In field, phorid flies were more likely to be found near disturbed ant mounds and/or trails. The flies of *P. litoralis*, *P. tricuspis*, and *P. wasmanni*, attacking ants near disturbed mounds, are expected to use alarm or defense compounds released by the ants as host location cues, and those of *P. borgmeieri*, *Pseudacteon nuicornis*, *P. obtusus*, and *Pseudacteon solenopsidis*, attacking near trails, are considered to use trail pheromone as a cue [60]. Therefore, workers are the primary source of long-range cues attracting phorid flies. Disturbance of fire ants causes the release of many defensive compounds, including alarm pheromone and venom alkaloids [61,62]. Electrical stimulation can be more effective in attracting phorid flies than mechanical mound disturbance presumably because it stimulates release of alarm pheromones and venom [63]. Defensive compounds other than alarm pheromones may also play an important role in this attraction since they are part of the alarm response and components of the complex alarm odor. Fire ant venom alkaloids prove to be key attractants for both male and female *P. tricuspis* [64]. Like the females, males of the phorid fly species are attracted to both fire ant alarm pheromone and venom alkaloids. Male flies of several species (e.g., *P. obtusus* and *P. tricuspis*) are also attracted to host ants apparently for finding mates [14]. The alarm pheromones shared by closely related ant species may serve as a general host location cue from long range, while the species-specific venom alkaloids are probably used as short-range cues for host location and host preference. The ratios of the *cis* and *trans* alkaloids and presence of some minor piperideine alkaloids [65,66], rather than abundance of the *cis* alkaloids, may be responsible for host preference by phorid fly for closely related ant species. Although alates contain a significantly higher concentration of the alarm pheromone than workers of *S. invicta*, *P. tricuspis* females rarely attack alates and are more attracted to worker ants compared to alates [7,67,68,69]. Fire ant mating flights often attract large numbers of phorid flies due to release of alarm pheromone triggered by alate activity [69,70,71].

## 6. Conclusions

Phorid flies in the genus *Pseudacteon* are highly specific parasitoids of ants. A female fly lays an egg into the thorax of a live worker ant, and the larva eventually decapitates the host ant after consuming all head tissues. Some flies prefer large workers, whereas others tend to attack small to medium-sized workers [32,37]. Furthermore, many *Pseudacteon* flies are sympatric, exhibiting different annual and daily activity patterns. In addition to the direct effect of mortality on their host ants, *Pseudacteon* phorids affect fire ant foraging behavior and have population-level impacts on the survival of the host ants [2]. The presence of *Pseudacteon* phorids may weaken the competiting vigor of the host species relative to other ant species in the community. Because of these direct and indirect effects, *Pseudacteon* phorids are a promising group for biological control of invasive *Solenopsis* fire ants.

## Figures and Tables

**Table 1:** Mean egg-larval, pupal, and total development times for female *Pseudacteon* flies reared on the imported *Solenopsis* fire ants at various temperatures.

Species	Temp. (°C)	Host Species	Development Time (d)			Reference
			Larval	Pupal	Total	
*P. borgmeieri*	22–27	*S. richteri*	26.0	30.0	56.0	[21]
		*S. invicta*	34.0	29.0	62.0	[21]
*P. cultellatus*	22–27	*S. richteri*	20.5	27.0	47.0	[21]
		*S. invicta*	19.0	23.0	42.0	[21]
*P. curvatus*	22–27	*S. richteri*	13.0	18.0	31.0	[21]
*P. litoralis*	23	*S. invicta*	22.0	24.0	47.0	[16]
	30	*S. invicta*	18.4	18.7	37.1	[23]
*P. nocens*	22–27	*S. richteri*	32.0	32.5	65.0	[21]
		*S. invicta*	25.0	26.5	51.5	[21]
*P. nudicornis*	22–27	*S. richteri*	20.0	19.0	42.0	[21]
		*S. invicta*	16.0	19.0	35.0	[21]
*P. tricuspis*	22–27	*S. richteri*	19.0	17.0	38.0	[21]
	24	*S. invicta*, hybrid	20.0	19.0	39.0	[19]
	30	*S. invicta*	15.9	17.2	33.1	[23]
*P. obtusus*	22–27	*S. richteri*	15.0	23.0	38.0	[21]
		*S. invicta*	22.0	27.0	49.0	[21]
